# Should We Rule out Celiac Disease in Recurrent Headache Disorders? A Review of the Literature

**DOI:** 10.3390/jcm13092615

**Published:** 2024-04-29

**Authors:** Lorenzo Perilli, Samanta Carbone, Luca Franco Novelletto, Andrea Santangelo, Maria Rosaria Curcio, Federica Lotti, Salvatore Grosso

**Affiliations:** 1 Clinical Pediatrics, Department of Molecular Medicine and Development, University of Siena, Azienda Ospedaliero-Universitaria Senese, 53100 Siena, Italy; 2 Pediatric Neurology, Department of Pediatrics, Santa Chiara Hospital, Azienda Ospedaliero Universitaria Pisana, 56126 Pisa, Italy; 3 Department of Neurosciences, Rehabilitation, Ophthalmology, Genetics, Maternal and Child Health, University of Genoa, 16132 Genoa, Italy

**Keywords:** headache, migraine, celiac disease, gluten-free diet

## Abstract

Recurrent headaches, encompassing migraine and tension-type headaches, represent prevalent conditions affecting individuals across different age groups, exerting a substantial influence on daily functioning and quality of life. Headaches serve as common manifestations of underlying health issues. Among these, celiac disease, an autoimmune disorder activated by gluten consumption, has emerged as a noteworthy concern. Recent research indicates a correlation between celiac disease and heightened susceptibility to headaches, particularly migraines. Celiac disease (CD) is an immune-mediated systemic, widespread disorder presenting a heterogeneous constellation of symptoms with a relatively easy diagnosis and therapy. Among signs and symptoms exhibited in celiac disease patients, headache is one of the most common neurological issues addressed among both adults and children. Headache disorders and CD are highly prevalent in the general population; for this reason, any causal association between these conditions and the role of a gluten-free diet (GFD) has been debated. The aim of this manuscript is to review the current scientific literature regarding the potential association between CD and headaches and the beneficial effects of a GFD. Among the various authors, in our opinion, the current state of the evidence suggests a significant role for the early screening of CD during the initial diagnosis of recurrent headaches, either in adults or children.

## 1. Introduction

Headaches are a ubiquitous affliction globally, exhibiting a wide spectrum of clinical characteristics and potential underlying causes. Among the diverse headache types, tension headaches, characterized by a dull, band-like pain, and migraines, marked by intense throbbing pain accompanied by additional symptoms like nausea and sensitivity to stimuli, are prevalent [1]. Other types include cluster headaches, sinus headaches arising from sinus inflammation or infection, hormone headaches, and rebound headaches.

Various etiologic factors contribute to the development of headache disorders, such as genetic and environmental. While many headaches are transient and harmless, they can also be indicative of more significant health issues, including celiac disease, an autoimmune disorder triggered by gluten ingestion. Recognizing the various headache types and their potential connection to celiac disease is pivotal for comprehensive healthcare management.

In recent years, celiac disease has garnered considerable scientific attention due to its widespread prevalence and heterogeneous symptom presentation, coupled with relatively straightforward diagnostic and therapeutic approaches. Research indicates that individuals with celiac disease may have an increased susceptibility to migraines and other headache types compared to the general population.

CD is an immune-mediated systemic disorder provoked by gluten and related prolamins in genetically susceptible subjects. It is characterized by the presence of a variable combination of gluten-dependent clinical manifestations, CD-specific antibodies, human leucocyte antigens (HLA-DQ2 or HLA-DQ8 haplotypes), and enteropathy [2].

Both innate and adaptive immune dysregulation contribute to its pathogenesis, leading to a predisposition for other autoimmune diseases based on HLA DQ2-DQ8, such as Hashimoto thyroiditis, type 1 diabetes mellitus, Addison’s disease, and autoimmune hepatitis [3]. Its prevalence in the general population ranges from 0.5 to 2%, with an average of about 1% [4]. In Italian school children, recent data from the literature has suggested that the prevalence in the last 25 years has doubled from 0.88 to 1.58% [5].

CD can present at any age, but it typically demonstrates two distinct peaks of incidence: the initial peak occurs within the first two years of life, following gluten exposure, while the second peak arises between the second and third decades [6]. Early detection of pediatric cases is imperative, not only for favorable long-term outcomes but also to ensure strict adherence to a gluten-free diet for effective therapeutic management.

In Europe, children can receive a biopsy-sparing diagnosis based on the latest 2020 ESPGHAN (European Society Pediatric of Gastroenterology, Hepatology and Nutrition) guidelines [2]. This diagnosis requires IgA-producing subjects to have a tenfold increase in anti-transglutaminase IgA (anti-tTG) and positivity for anti-endomysium antibodies (EMA) in a subsequent test, even in the absence of other symptoms. Currently, non-invasive alternatives, such as radioimmunological detection of anti-tTG in saliva samples, are under investigation [7]. In adults, a biopsy confirmation is always necessary to confirm diagnosis.

The presenting symptoms of CD vary from diarrhea, failure to thrive, and malabsorption. However, in recent years, these features have declined as diagnostic criteria and expertise have improved. Nowadays, fatigue, constipation, headache, nausea, iron deficiency anemia, and joint pain can be recognized as isolated extra-intestinal complications of “atypical” CD. Among these signs and symptoms, headache is one of the most common neurological issues experienced by both adults and children. In the pediatric and adult populations, headache disorders (headache, migraine, and tension-type headache) have a significant impact on daily activities.

Approximately 1 in 10 children report recurrent headaches due to migraines, leading to significant impairment in school performance and quality of life [8]. The prevalence of tension-type headaches in the pediatric population varies widely, ranging from 29% to 71% [9,10]. Recent studies in the adult US population indicate a prevalence of migraine and severe headache affecting roughly 1 out of every 6 Americans and 1 in 5 women over a 3-month period [11].

Various limitations in worldwide data collection have been reported [12,13]. Among other neurological symptoms reported at the onset of “atypical” celiac disease patients, such as cerebellar ataxia, peripheral neuropathies, and epilepsy [14], headache disorders have been frequently reported. However, their association with CD has been extensively debated in scientific literature. Additionally, various authors have emphasized the significant role of a gluten-free diet in reducing the frequency and intensity of headaches in newly CD-diagnosed patients [14,15,16,17,18,19,20]. Our aim is to delve into the complex topic of the risk of headaches in CD patients and vice versa, providing insights for clinicians handling diagnoses in patients with recurrent headaches.

## 2. Materials and Methods

### 2.1. Search Strategy

This search is reported in accordance with the Preferred Reporting Items for Systematic Reviews and Meta-Analysis (PRISMA) guidelines. A search of case-control studies investigating the association between headache disorders and CD was performed in February 2024. Two medical subject headings (MeSH) were used: Term A was celiac disease, and Term B was headache. Only English-published material was included. The reference lists of included articles were examined in order to identify further relevant articles.

### 2.2. Inclusion and Exclusion Criteria

- Articles included in the present review met the following inclusion criteria:
- Articles published between 2002 to 2024.
- Case-control studies or other articles containing utilizable information investigating the association between CD and headache disorders.
- English language article.

The search strategy identified 31 articles. A total of 25 articles were excluded during the selection. Upon examination of the reference lists of the studies that were included, we found 10 more articles that met our criteria for inclusion. These articles were not previously found in the search strategy mentioned earlier. A total of 16 articles were reviewed.

**Figure 1:**
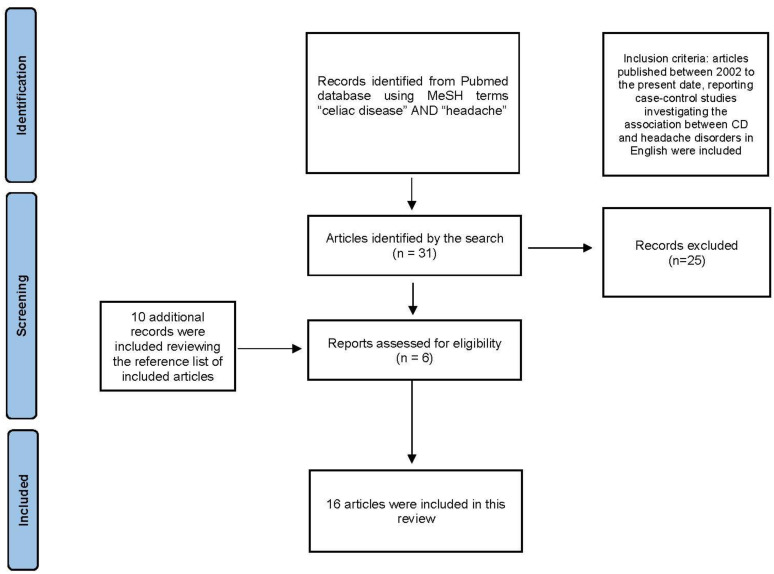
Preferred Reporting Items for Systematic Reviews and Meta-Analysis (PRISMA) chart.

Figure 1 illustrates the PRISMA chart describing the selection process.

## 3. Results

### 3.1. Association between Headache, Migraine and Celiac Disease

Numerous studies have investigated the potential association between headache disorders and celiac disease. The collective evidence suggests a consistent trend towards a higher prevalence of headaches in CD patients compared to the general population and vice versa. This association remains robust even among CD patients who lack histological bowel alterations, indicating that factors beyond intestinal damage may contribute to headache pathogenesis in CD.

Several retrospective case-control studies, such as those led by Zelnik et al. [15], have unveiled a significant rise in headache prevalence among children and young adult CD patients compared to the control group. The authors noted headache as the most prevalent neurological disorder in CD patients, predominantly comprising migraines, followed by tension-psychogenic headaches. However, a notable limitation of this study lies in the variability of diagnostic criteria during enrollment, potentially leading to an underestimation of real CD prevalence.

Hom et al. confirmed these findings through a retrospective study, emphasizing a heightened prevalence of headaches, particularly migraines, within the CD children population [16]. The prevalence of headache disorders was observed to be higher in individuals with a familial history of headaches, earlier age of diagnosis, and familial history of CD.

Additionally, Lionetti et al., in a retrospective case-control study, reported a heightened prevalence of pediatric CD patients experiencing headaches, primarily bilateral, before diagnosis [14]. This finding was confirmed in a subsequent phase of the study. Moreover, Ameghino et al. reported that in a large cohort of CD patients, 24% presented with headaches as the principal symptom that resulted in diagnosis, predominantly tension-type headaches followed by migraines [21]. However, this study is limited by its reliance on self-reported data and inclusion of only histologically confirmed CD patients with headaches.

Supporting an organic impact of CD on brain activity, Parisi et al. reported the prevalence of electroencephalographic (EEG) abnormalities in 47.4% of the CD patients, with 36.8% experiencing headaches affecting daily activities [17].

In the adult population, Gabrielli et al. found a higher percentage of positive CD serology in migraine patients compared to controls (4.4% vs. 0.4% *p* < 0.05) [22].

Notably, Lahat et al. reported no increased prevalence of CD in pediatric patients with migraines. However, this study solely relied on anti-gliadin IgA/IgG and anti-endomysium tests, as EGDS was not performed due to the negativity of EMA, which could lead to underestimation. In fact, recent literature highlighted unaltered EMA in potential CD populations with persistent low titer of anti-tTG [23,24]. Moreover, in the latest ESPGHAN guidelines, anti-gliadin IgG dosage is not recommended, even in children under two years old [2].

Contrastingly, Alehan et al. highlighted an increased prevalence of positive serum CD in the migraine headache study group: total IgA and anti-tTG IgA were dosed, and serum CD antibodies were found positive (5.5% against 0.6% in the control group, *p* = 0.043) [25]. The biopsy results were negative for either the study or control group. The lack of EMA analysis and the use of an ELISA kit, which has less sensitivity than chemiluminescence or RIA, can represent a mild limitation.

Borgna Pignatti et al. observed a similar trend of elevated prevalence of celiac disease (CD) within a cohort of pediatric patients suffering from migraine [26]. Notably, distinct methodologies for celiac disease serum testing were employed across the two cohorts: the study group underwent testing for anti-tTG IgG, whereas the control group was assessed for EMA IgA and anti-gliadin IgA/IgG, being part of a previous study. The authors asserted that despite these methodological differences, data were deemed comparable due to reported overall accuracy between EMA and anti-tTG assays. However, the study did not report the assessment of anti-tTG IgA levels in the study group. Notably, current European pediatric guidelines regard anti-tTG IgG as a non-specific marker for CD diagnosis. Consequently, in individuals with immunoglobulin A deficiency, a tenfold elevation in anti-tTG IgG levels necessitates histological confirmation to ascertain CD diagnosis. Additionally, as mentioned above, this correspondence has not been demonstrated in the study by Trovato et al. [24].

In the study conducted by Inaloo et al., the prevalence of celiac disease was investigated among pediatric patients diagnosed with migraine, juxtaposed with a control cohort comprising healthy individuals [27]. Using measurements of total IgA and anti-tTG IgA, the researchers assessed 100 pediatric migraine patients and 1500 healthy controls. Surprisingly, no significant difference in CD prevalence was discerned between the two groups, both exhibiting a prevalence rate of 2%. Notably, EMA were not dosed, and among those diagnosed with CD, an improvement in migraine symptoms was observed upon adherence to a gluten-free diet after histologic confirmation.

Similarly, Dimitrova et al. examined the prevalence of migraine in patients with CD, inflammatory bowel disease (IBD), gluten sensitivity, and the general population [18]. Their findings unveiled a heightened prevalence of migraine within the CD patient group, with migraine being the most frequently reported headache disorder, followed by chronic headaches and tension-type headaches. It is pertinent to note that data acquisition in this study relied on self-reported information, with the CD diagnosis based on positive serological tests and/or biopsy results.

Furthermore, Lebwohl et al. conducted an extensive case-control analysis encompassing 28,638 CD patients, revealing a predominant occurrence of headache-related visits among this demographic. Specifically, the presence of migraine was significantly linked with a prior diagnosis of CD, denoted by a hazard ratio (HR) of 1.67 and a 95% confidence interval (CI) spanning 1.48–1.87, indicative of a robust association [28].

Moreover, Nenna et al. elucidated a higher prevalence of CD among children and adolescents afflicted with headache disorders relative to the general populace. Among the study group, 11 individuals (1.25%) exhibited positive serological indicators (total IgA, anti-tTG IgA, and EMA antibodies) alongside confirmatory biopsy results, while seven patients (0.79%) were already diagnosed with CD at the first neurological evaluation [29]. These results showed that celiac disease prevalence is doubled in patients with recurrent headaches (2.04% vs. 1.2%; *p* = 0.034), suggesting CD screening as part of the diagnostic work-up in pediatric patients, particularly among pharmacological non-responders. As observed across most studies, migraine emerged as the predominant disorder, followed by tension-type headaches.

### 3.2. Aetiology and Pathogenesis

The exact mechanisms underlying the association between CD and headache disorders remain the subject of ongoing research, but several hypotheses have been proposed. One prominent etiopathogenetic hypothesis implicates pro-inflammatory cytokines in the pathophysiology of both CD and primary headache disorders, particularly migraine [30]. Interleukin 1β and tumor necrosis factor alpha, well-established players in the pathophysiology of migraine, are often elevated in the serum of migraine sufferers [31]. Calcitonin gene-related peptide (CGRP), which has been demonstrated to be secreted during migraine attacks and induced by cytokines, is found in over half of the neurons in the trigeminal ganglion. In this region, the cytokines activate the trigeminal nerve, hence transmitting pain signals [32]. Trigeminovascular activation that is repeated and persistent can sensitize brainstem nuclei, leading to a state of central sensitization. In essence, migraine pathophysiology encompasses a cascade of processes, starting with neurogenic inflammation, followed by peripheral trigeminovascular input, and culminating in the activation of central cortico-trigeminal nuclei.

In this context, CD could theoretically contribute to cytokine circulation and, hence, to the pathogenesis of headaches. In fact, in CD patients, the immune system overreacts to gluten and ultimately induces a T-cell-mediated autoimmune enteropathy and inflammatory response. Hence, the increased concentrations of interferon-gamma and TNF-α, which control CGRP synthesis, may explain the association between migraine and celiac disease.

Another common aspect of both celiac disease and headache disorders is the well-known association between dysbiosis and the gut–brain axis. In fact, some authors hypothesized that the “nutritional–microbial–epithelial–neuronal” akin to “environmental–luminal–mucosal–neuronal” brain network could be the cause for the extra-intestinal manifestations of celiac disease [33]. Additionally, gut dysbiosis contributes to the pro-inflammatory milieu by facilitating the leakage of lipopolysaccharides (LPS) into the circulation [30].

The onset and exacerbation of headaches in celiac disease seem to be closely linked to immune-mediated inflammatory responses triggered by gluten ingestion. Gluten peptides can activate the immune system, leading to a cascade of pro-inflammatory cytokines and chemokines. This inflammatory storm contributes to altering the blood–brain barrier integrity, allowing cytokines induced by gluten to infiltrate the brain parenchyma, triggering headaches through neuroinflammatory pathways [34,35].

While genetic factors have been investigated, no clear correlation has been established between migraine and CD [31]. However, recent studies have highlighted the potential role of circulating autoantibodies to Transglutaminase 6 in predisposed individuals to neurological disorders, including headaches [36]. In light of these findings, exploring the interplay between pro-inflammatory cytokines, autoimmune reactions, and genetic predisposition may provide valuable insights into the link between CD and headache disorders.

### 3.3. Role of the Gluten-Free Diet

The gluten-free diet is the only known therapy for celiac disease patients. Absolute compliance results in the negativization of autoantibody level and the disappearance of both extra and gastrointestinal-related symptoms.

The therapeutic efficacy of a gluten-free diet (GFD) in the management of headaches among individuals with celiac disease (CD) has attracted substantial scientific interest. Although it has been frequently reported in the literature and shown to be of significant importance in newly diagnosed CD patients, limitations in the precedent studies and changes in guidelines make it difficult to assess research results and the real impact.

Studies conducted by Lionetti [14], Zelnick [15], Hadjivassiliou [19], and Ciccarelli et al. [20] have consistently shown that adherence to a strict GFD is associated with improvements in headache symptoms. Some authors have highlighted consistent results throughout a 1-year and 7-year follow-up [19]. This suggests a potential causal link between gluten exposure and headache pathophysiology in susceptible individuals.

Hom et al. corroborated these findings. Their study revealed that more than two-thirds of the participants who noted headaches before a confirmed diagnosis of CD experienced improvements in headache frequency or both frequency and intensity following adherence to a gluten-free diet [16].

The impact of GFD adherence on headache outcomes appears to be influenced by the degree of compliance. Patients with optimal adherence to a GFD demonstrate sustained improvement or resolution of headaches over time, whereas those with suboptimal adherence continue to experience symptoms, as highlighted by Hadjvassiliou [19].

Further insights from Parisi et al. observed that a significant percentage of CD patients with EEG abnormalities experienced headaches affecting daily activities, with many showing 77.7% resolution of these abnormalities after adopting a GFD [17].

In the adult population, Dimitrova et al. reported that a gluten-free diet (GFD) did not demonstrate protective effects against the onset of migraine [18]. However, it is possible that this evidence might be underestimated due to the survey’s limitation. The authors underscored that subjects were only asked to report current headaches, thus lacking detailed clinical information and potentially underestimating improvements in headache symptoms following the initiation of GFD. Furthermore, compliance with GFD was not assessed. Additionally, eight CD patients reported improvements in symptoms or even complete resolution of migraine episodes, entering their comments as free text.

Lastly, Nenna et al. reported that the initiation of a gluten-free diet was significantly effective in newly diagnosed patients, resulting either in the complete disappearance or consistent improvements of headache symptoms [29].

The therapeutic efficacy of a GFD in ameliorating headache symptoms further underscores the importance of recognizing and addressing CD in patients with recurrent or refractory headache symptoms.

## 4. Discussion

The state of the evidence suggests a statistical association between celiac disease and headache disorders, two highly prevalent and widespread conditions [4,5,6,7,8,9,10,11,12,13,14,15,16,17,18,19,20,21,22,23,24,25,26,27,28,29,30,31,32,33,34,35,36,37]. Reviewing the current literature on this topic, it emerges clearly that, despite the high incidence of both these diseases, studies have highlighted an increased prevalence of CD among headache disorder patients and a significant effect of GFD on recurrent cephalgia.

In a systematic review and meta-analysis, Zis et al. highlighted that the prevalence of headaches in patients with CD was about 26% and 18.3% in adult and pediatric populations, respectively, and was the initial presenting symptom of CD. It is also reported that after GFD, up to 75% of patients diagnosed with CD had total resolution of headache [38].

All things considered, there are some doubts: Should we routinely test patients experiencing recurrent and/or intractable headache disorders for celiac disease? The impact of headaches in children and adolescents’ daily lives (migraine and tension-type headaches) account for 7% and 37.5%, respectively, among all causes of Years Lived with Disability (YLDs) [37]. In clinical practice, this knowledge is often not considered. Most of the time, headache patients who refer to physicians, even if reporting a frequent need for pharmaceutical therapy, undergo blood tests only after numerous examinations. At least in the pediatric population, 2020 ESPGHAN guidelines indicate that CD should be serologically diagnosed, even in asymptomatic patients [2], so considering this recommendation and the association between headache disorders and CD, excluding it in the initial diagnostic should be mandatory when such symptoms are reported.

However, the limitations presented in the studies gives food for thought for the necessity for CD screening in intractable/recurrent headache patients.

A difficult bias to overcome when comparing results is the limitations regarding changes in guidelines, advances in knowledge, and data availability. This element likely makes data not fully reliable, underscoring both CD prevalence among migraineurs and GFD efficacy. Data regarding pharmaceutical treatment in most of the studies reviewed are incomplete. Lebwhol et al. [28] stated that the lack of information about NSAIDs in the enrolled cohort could act as a limitation. Some other authors [14,22,23] mentioned that newly CD-diagnosed patients were instructed to take anti-inflammatory drugs in case of headache after a GFD was started.

Interestingly, among the CD study group, Nenna mentioned a patient in which triptans, administered as anti-migraine prophylactic therapy, were later suspended after GFD initiation; similarly, 10 patients stopped NSAIDs after diet therapy.

The authors further hypothesized that the recovery of the intestinal mucosa could eventually contribute to the effectiveness of drug therapy. The possible influence of NSAIDs on migraine in the cohort was collaterally investigated by Dimitrova et al. [18], who found no significant association (*p* = 0.3772). This evidence is not considered meaningful because of the low number of patients who reported taking medications.

Further studies are needed to determine whether all recurrent/intractable cephalgic patients or a selected sub-group should be tested for CD and the impact of a GFD on headache symptoms in light of the recent findings about both these diseases.

Collaborative efforts between healthcare providers, including neurologists and gastroenterologists, are crucial in addressing both the gastrointestinal and neurological aspects of celiac disease and associated headaches.

## 5. Conclusions and Future Directions

Celiac disease appears to be strictly related to headache disorders and vice versa, even if this evidence is still debated in the scientific community, given the high prevalence, the commonness of both diseases and the limitations in reported data. We addressed such a peculiar topic because, in our experience as an Italian pediatric neurology study group, considering the high prevalence of CD in our territory, celiac disease is often tested due to the heterogeneity of its extra-intestinal symptoms. Autoimmunity, hypercytokinemia, and metabolic deficiencies caused by malabsorption have been indicated as possible etiopathological causes for the increased prevalence of neurological symptoms encountered among CD patients. On the one hand, strict adherence to a gluten-free diet showed improvement or, in some cases, complete resolution of headache disorders; on the other hand, persistence of the symptoms was noted in GFD non-compliant patients. Moreover, it must be considered that this evidence persisted over the years and through the changes that diagnostic guidelines underwent, corroborating these correlations. Considering the most recent evidence about celiac disease, most of the reported data are only partially reliable. 

Further studies are needed on this topic, but in our opinion, considering the current literature, the small cost of serology, and the potentially great impact on patients’ lives, CD screening should be part of the initial diagnostic work-up, among other routine blood tests when approaching a patient with recurrent/intractable headache or migraine.

## Data Availability

The data supporting the conclusions of this article will be made available by the authors without undue reservation.

## References

[1] Liu A. (2021). Headaches. Pediatr. Ann..

[2] Husby S., Koletzko S., Korponay-Szabó I., Kurppa K., Mearin M.L., Ribes-Koninckx C., Shamir R., Troncone R., Auricchio R., Castillejo G. (2020). European Society Paediatric Gastroenterology, Hepatology and Nutrition Guidelines for Diagnosing Coeliac Disease 2020. J. Pediatr. Gastroenterol. Nutr..

[3] Caio G., Volta U., Sapone A., Leffler D.A., De Giorgio R., Catassi C., Fasano A. (2019). Celiac disease: A comprehensive current review. BMC Med..

[4] Catassi C., Verdu E.F., Bai J.C., Lionetti E. (2022). Coeliac disease. Lancet.

[5] Gatti S., Lionetti E., Balanzoni L., Verma A.K., Galeazzi T., Gesuita R., Scattolo N., Cinquetti M., Fasano A., Catassi C. (2020). Increased Prevalence of Celiac Disease in School-age Children in Italy. Clin. Gastroenterol. Hepatol..

[6] Rubin J.E., Crowe S.E. (2020). Celiac disease. Ann. Intern. Med..

[7] Nenna R., Tiberti C., Luparia R.P.L., Perricone C., Lucantoni F., Masotti D., Mennini M., Montuori M., Bonamico M. (2008). 5000 six to eight year-old Italian school-children screened for coeliac disease using salivary RIA anti-transglutaminase antibody detection. Dig. Liver Dis..

[8] Robbins M.S., Szperka C. (2021). Headache in Children and Adolescents. Contin. Lifelong Learn. Neurol..

[9] Göbel H., Petersen-Braun M., Soyka D. (1994). The Epidemiology of Headache in Germany: A Nationwide Survey of A Representative Sample on The Basis of The Headache Classification of The International Headache Society. Cephalalgia.

[10] John S., Hajj-Ali R.A. (2014). Headache in Autoimmune Diseases. Headache J. Head Face Pain.

[11] Burch R., Rizzoli P., Loder E. (2018). The Prevalence and Impact of Migraine and Severe Headache in the United States: Figures and Trends From Government Health Studies. Headache J. Head Face Pain.

[12] Victor T., Hu X., Campbell J., Buse D., Lipton R. (2010). Migraine prevalence by age and sex in the United States: A life-span study. Cephalalgia.

[13] Onofri A., Pensato U., Rosignoli C., Wells-Gatnik W., Stanyer E., Ornello R., Chen H.Z., De Santis F., Torrente A., Mikulenka P. (2023). Primary headache epidemiology in children and adolescents: A systematic review and meta-analysis. J. Headache Pain.

[14] Lionetti E., Francavilla R., Maiuri L., Ruggieri M., Spina M., Pavone P., Francavilla T., Magistà A.M., Pavone L. (2009). Headache in Pediatric Patients with Celiac Disease and Its Prevalence as a Diagnostic Clue. J. Pediatr. Gastroenterol. Nutr..

[15] Zelnik N., Pacht A., Obeid R., Lerner A. (2004). Range of Neurologic Disorders in Patients with Celiac Disease. Pediatrics.

[16] Hom G.L., Hom B.L., Kaplan B., Rothner A.D. (2019). A Single Institution’s Experience of Primary Headache in Children With Celiac Disease. J. Child Neurol..

[17] Parisi P., Pietropaoli N., Ferretti A., Nenna R., Mastrogiorgio G., Del Pozzo M., Principessa L., Bonamico M., Villa M.P. (2015). Role of the gluten-free diet on neurological-EEG findings and sleep disordered breathing in children with celiac disease. Seizure.

[18] Dimitrova A.K., Ungaro R.C., Lebwohl B., Lewis S.K., Tennyson C.A., Green M.W., Babyatsky M.W., Green P.H. (2013). Prevalence of Migraine in Patients with Celiac Disease and Inflammatory Bowel Disease. Headache J. Head Face Pain.

[19] Hadjivassiliou M., Croall I.D., Grünewald R.A., Trott N., Sanders D.S., Hoggard N. (2021). Neurological evaluation of patients with newly diagnosed coeliac disease presenting to gastroenterologists: A 7-year follow-up study. Nutrients.

[20] Cicarelli G., Della Rocca G., Amboni M., Ciacci C., Mazzacca G., Filla A., Barone P. (2003). Clinical and neurological abnormalities in adult celiac disease. Neurol. Sci..

[21] Ameghino L., Farez M., Wilken M., Goicochea M. (2019). Headache in Patients with Celiac Disease and Its Response to the Gluten-Free Diet. J. Oral Facial Pain Headache.

[22] Gabrielli M. (2003). Association between migraine and celiac disease: Results from a preliminary case-control and therapeutic study. Am. J. Gastroenterol..

[23] Lahat E., Broide E., Leshem M., Evans S., Scapa E. (2000). Prevalence of celiac antibodies in children with neurologic disorders. Pediatr. Neurol..

[24] Trovato C.M., Montuori M., Morelli A., Fegatelli D.A., Vestri A., Giordano C., Cucchiara S., Caio G., Oliva S. (2021). Diagnostic value of persistently low positive TGA-igA titers in symptomatic children with suspected celiac disease. J. Pediatr. Gastroenterol. Nutr..

[25] Alehan F., Ozçay F., Erol I., Canan O., Cemil T. (2008). Increased Risk for Coeliac Disease in Paediatric Patients with Migraine. Cephalalgia.

[26] Borgna-Pignatti C., Fiumana E., Milani M., Calacoci M., Soriani S. (2004). Celiac Disease in Children with Migraine. Pediatrics.

[27] Inaloo S., Dehghani S.M., Farzadi F., Haghighat M., Imanieh M.H. (2011). A comparative study of celiac disease in children with migraine headache and a normal control group. Turkish J. Gastroenterol..

[28] Lebwohl B., Roy A., Alaedini A., Green P.H.R., Ludvigsson J.F. (2016). Risk of Headache-Related Healthcare Visits in Patients with Celiac Disease: A Population-Based Observational Study. Headache J. Head Face Pain.

[29] Nenna R., Petrarca L., Verdecchia P., Florio M., Pietropaoli N., Mastrogiorgio G., Bavastrelli M., Bonamico M., Cucchiara S. (2016). Celiac disease in a large cohort of children and adolescents with recurrent headache: A retrospective study. Dig. Liver Dis..

[30] Musubire A.K., Cheema S., Ray J.C., Hutton E.J., Matharu M. (2023). Cytokines in primary headache disorders: A systematic review and meta-analysis. J. Headache Pain.

[31] Hadjivassiliou M., Croall I.D., Zis P., Sarrigiannis P.G., Sanders D.S., Aeschlimann P., Grünewald R.A., Armitage P.A., Connolly D., Aeschlimann D. (2019). Neurologic Deficits in Patients with Newly Diagnosed Celiac Disease Are Frequent and Linked with Autoimmunity to Transglutaminase 6. Clin. Gastroenterol. Hepatol..

[32] Qasim H., Nasr M., Mohammad A., Hor M., Baradeiya A.M. (2022). Dysbiosis and Migraine Headaches in Adults with Celiac Disease. Cureus.

[33] Arzani M., Jahromi S.R., Ghorbani Z., Vahabizad F., Martelletti P., Ghaemi A., Sacco S., Togha M., School of Advanced Studies of the European Headache Federation (EHF-SAS) (2020). Gut-brain Axis and migraine headache: A comprehensive review. J. Headache Pain.

[34] Wiggers A., Ashina H., Hadjikhani N., Sagare A., Zlokovic B.V., Lauritzen M., Ashina M. (2022). Brain barriers and their potential role in migraine pathophysiology. J. Headache Pain.

[35] Biscetti L., Cresta E., Cupini L.M., Calabresi P., Sarchielli P. (2023). The putative role of neuroinflammation in the complex pathophysiology of migraine: From bench to bedside. Neurobiol. Dis..

[36] Stovner L.J., Hagen K., Linde M., Steiner T.J. (2022). The global prevalence of headache: An update, with analysis of the influences of methodological factors on prevalence estimates. J. Headache Pain.

[37] Leonardi M., Grazzi L., D’Amico D., Martelletti P., Guastafierro E., Toppo C., Raggi A. (2020). Global Burden of Headache Disorders in Children and Adolescents 2007–2017. Int. J. Environ. Res. Public Health.

[38] Zis P., Julian T., Hadjivassiliou M. (2018). Headache Associated with Coeliac Disease: A Systematic Review and Meta-Analysis. Nutrients.

